# Reviews of Books

**Published:** 1923-08

**Authors:** 


### REVIEWS OF BOOKS.

### THE CONQUEST OF MALARIA.

**Memoirs : With a Full Account of the Great Malaria Problem and Its Solution.** By RONALD ROSS, C.B., K.C.B., K.C.M.G., F.R.S., F.R.C.S., M.D., etc. (Murray. 24s. net.)

Poet, author, mathematician, surgeon and bacteriologist, Sir Ronald Ross has many qualifications for dealing with a scientific subject. Gifted with the gladiatorial spirit, with a taste for tilting at all and sundry, he has given his readers a thrilling account of a running fight full of incident and colour. He has assuredly chosen wisely in addressing his Memoirs to the general reader as well as to medical men. His style is too florid for a strictly medical palate, and it would have been a vast pity had this record of a romantic career been available only to the author's colleagues. After all, it is the layman, not the doctor, who rules the world, and the more laymen are familiarised with the problems and difficulties of the scientist, the better it will be for science and humanity.

This big book contains such an odd assortment of gossip, anecdotes, philosophical essays, poetry and other diversions that the reader will feel grateful to the author for the instructive headings to his chapters. Such titles as "The Morning Stars," "False Scents," and "The Discovery" not only whet the appetite, but help the reader to penetrate easily to the most thrilling episodes. It is not till nearly the middle of the book that "The Discovery" is described. The description does not suffer from lack of a lurid setting. Here is a sample :—

> I did not allow the punka to be used because it blew about my dissected mosquitoes, . . . and the result was that swarms of flies and of "eye-flies"—minute little insects which try to get into one's ears and eyelids—tormented me at their pleasure, while an occasional *Stegomyia* revenged herself on me for the death of her friends. The screws of my microscope were rusted with sweat from my forehead and hands, and its last remaining eye-piece was cracked !

On August 22, 1897, the author wrote to his wife :—"I really think I have done the mosquito theory at last, having found something in mosquitoes fed on malaria patients exactly like the malaria parasite." These were thrilling times to which a tragi-comedy, an exasperating anti-climax, was to be added. Sir Ronald received, on September 24, instructions from Headquarters to proceed immediately to Bombay for military duty. He writes :—"Owing to no fault of mine, two long years were to elapse before I was to see again—in another Continent—that wonderful revelation of human malaria in mosquitoes. During that time all my work was to be pirated by foreigners, and the same maladministration which now drove me into the wilderness was to force me finally out of India just when my discovery might have been of real help to her swarming and dying millions." He was never able to ascertain by whom, and for what purpose, he was thus peremptorily driven into the wilderness, and when he wired to the Principal Medical Officer, Bombay, he received the answer :—"Nothing known here about you." Looking back on these events, the author may perhaps find consolation in the justifiable reflection that this message was more applicable to the sender than the receiver.

The years which followed the discovery of 1897 were thrilling years of attempted piracy and brigandage. Scientific thieves are thus described in the chapter on Roman Brigandage :—

> When some patient investigator has announced some new fact they repeat a few of his investigations—which with the abundance of material they possess is generally an easy matter—and then, without waiting to corroborate details, begin to pour forth a flood of literature, in order to render it more credible. No consecutive history or exhaustive bibliography is given, and there are many old hypotheses which appear to be new and brilliant to the majority of readers. . . . Dr. Calandruccio, himself a victim, remarked that though his countrymen had succeeded in putting down brigandage under arms, they had introduced a new kind of brigandage in its place which consists of robbery in science.

Fortunately Sir Ronald had many friends, and after 1900 honours and recognition flowed in on a strong tide. About noon on Monday, November 3, 1902, he found a registered letter in his laboratory. It was from Stockholm and ran :—"I have the honour to inform you that the Professorial Staff of the Caroline Medico-surgical Institute in Stockholm has this day decided to awarde to you the medical Nobelprize of this year in amount of 141,846 Sweedish kroner (about 7,880£) for your work upon the Malaria." This decision was to have been kept a secret till December 10 of the same year, but Sir Ronald telegraphed the good news to his wife as "strictly private," and communicated it to certain friends on the same condition. When he entered his club about an hour after the receipt of this letter, he was the recipient of congratulations from every quarter, and in a day or two the secret was in the papers. He went to Stockholm to receive the Nobel Prize, and the account he gives of Swedish hospitality suggests that, had it lasted much longer, he would not have lived to write these highly entertaining Memoirs.

### THE MOTE AND THE BEAM.

**Visual Illusions : Their Causes, Characteristics and Applications.** By M. LUCKIESH. Illustrated. (Constable.)

We could have wished that the author of the adage, "Seeing is believing," had been put down to read this book before he gave to the world his famous dictum ; he would, we think, have been bound to agree with Luckiesh that seeing is at times very deceptive. Had the author written such a book a few hundred years ago, we fear that he would have risked being burnt as a most notorious wizard, while his book would probably also have been burnt by the public hangman and we should have lost a very fascinating work.

The author's aim has been to confine himself as much as possible to static visual illusions, to avoid an excess of theory, to keep as far as possible a non-controversial attitude towards his subject and to emphasize experimental facts, and in the course of the book he deals with geometrical illusions, equivocal figures, the influence of angles, illusions of depth and distance, irradiation and brightness-contrast, colour, lighting, painting and decoration, architecture, mirror magic and camouflage—a sufficiently varied table of contents. The opening chapters are very well done, the descriptions given being sufficient to enable even an unscientific person to grasp the essential facts of vision, and those which deal with many varieties of visual illusions are valuable not only for the examples quoted, but for the many experiments given which the reader can construct for himself. But all this, although it forms the main body of the book, forms an admirable introduction to what is really the most interesting section, particularly that on camouflage, the practical application of this subject to defence work in the late War.

The author tells us in his preface that " some theoretical aspects of the subject are still extremely controversial, so they are only introduced occasionally, and then chiefly for the purpose of illustrating the complexities and the trends of attempted explanations." He explains, too, that " the visual illusions discussed are chiefly of the static type, although a few others have been introduced. Some of the latter border upon motion, others upon hallucinations, and still others produced by external optical media are illusions only by extension of the term. These exceptions are included for the purpose of providing glimpses into the borderlands." We seem to reach these borderlands when the author refers to a possible illusion in the query of the Psalmist: " Why hop ye so, ye hills ? " while an amusing story is told of an old negro who shot several times without effect at what he took to be a squirrel on a tree trunk. His failure to make a kill was due to the fact that the squirrel was a louse on his eyebrow !

### MEALS FOR THE MALE.

**Diet for Men.** By Cecil Webb-Johnson, M.B., Ch.B. (Mills & Boon: 5s. net.)

The author has written with earnestness and sincerity on the evils of over-feeding, and especially the evils arising from the excessive consumption of flesh-foods, of alcohol and of tobacco. " Most people," he reminds us, " dig their graves with their teeth, and more people die from over-eating than over-drinking." He supplies useful figures to show the amount of food required by people in various occupations; but he says, " All scientists agree that the average Englishman eats about three times as much food as he needs," and " too much attention is paid to calories and vitamins." These assertions will hardly stand investigation, for modern science knows that the food consumed should be in proportion to the work to be done; that the energy spent in work is best expressed in calories or heat-units; and that the fuel intake should be proportionate to the calories expended. That some people exceed their actual necessity is no proof that all people are gluttons; indeed, many people are so much underfed that their condition is a matter of grave concern.

The pros and cons of " a mixed diet " are discussed, and a very useful list of " dont's " given. Some of Mr. Webb-Johnson's opinions would not be acceptable to the majority of medical men. He says that no one can enjoy good health who eats meat three times a day; this is contrary to experience. Among the evils of meat-eating he instances excessive irritability and bad temper. It is true that the flesh-eater is more lively, more energetic, responds more quickly to any stimulus, mental or physical, and as a rule is a better worker than the vegetarian. When a man has fixed the daily amount of food which is proper for him, it matters not, from a dynamic point of view, whether he eats it in one or three meals. But let us set this problem in dietetics: If a man takes no breakfast (as the author recommends), and only " a banana with a handful of almonds or raisins " for lunch (which he also recommends), how much food must he eat for dinner to gives the 2,500 calories of energy required by a man doing light work? The answer would show that the dinner would be too much for the stomach of the average man; and if he does not eat enough to supply the calories he is courting disaster.

Although the author says he is not a vegetarian, he has much to say in favour of vegetarianism. He lauds vegetarian sources of protein such as nuts, peas, beans; but he does not tell us that it is necessary to eat a greater proportion of them to get the same amount of digestible protein. Mr. Webb-Johnson declares that " eggs and vegetables, fresh fruit and cooked vegetables, and milk and vegetables, are bad combinations," but he gives no reason. Other doctors find that poached eggs and asparagus, or scrambled eggs and spinach make a very light and easily digested meal. What about eggs in a salad and mayonaise sauce ? What of dessert after an ordinary meal ? If milk and vegetables together are bad, what about the milk pudding after meat and vegetables ? His argument does not bear investigation.

Mr. Webb-Johnson argues against milk being taken by adults, and gives reasons for his opinions, which are not altogether acceptable. Moreover, he says it is not good for a patient with abnormal temperature ta take milk or other food, and " total abstention from food till the temperature is nearly normal guarantees a quicker recovery and lower death-rate." He advocates the consumption of water and fruit juice only when the temperature is high. This treatment might be efficacious in well-nourished people during a short rise of temperature; but it might become a dangerous rule if always adopted, for it should never be overlooked that heart-failure may occur from temporary removal of needed nourishment, and also that the energy used by the body in acute diseases is not less than 1,600 calories a day, and most people require food to supply it. It might be added that one pint of milk yields about 410 calories. There is much in the book with which we cannot agree, but it is readable enough, especially to those needing a popular exposition of the subject.

### THE EVOLUTION OF MEDICINE.

**The History of Medicine in its Salient Features.** By Walter Libby, M.A., Ph.D. Illustrated. (Constable: 15s.)

The history of the growth of a science plays the same part in this particular domain of knowledge as the spectroscope in astronomy, for it enables a determination of what may be described as the historic parallax, without which we cannot truly appreciate the cosmogony of facts and theories which make up the body of the individual science or determine their relationship with other sciences. But history plays another part. There is a determinism in the progress of knowledge as strict as that which exists in the physical world, and each new idea is the direct outcome of a chain of previous antecedent ideas stretching far into the distant past. No one can be thoroughly conversant with his subject who is not acquainted with its history.

From its intimate association with the first necessity of man—health—this is especially true of the history of medicine. Dr. Libby's book originated as a series of lectures given by the author to the third-year students in an American University. He treats his subject from a standpoint which is somewhat different from that adopted in many histories of medicine, by making it largely biographical, and by taking seriatim the development of various branches of medical science—embryology, cellular pathology, anæsthetics, antiseptic surgery and the like. The story is brought down to the present time by the inclusion of chapters upon the history of syphilis and of malaria, and a chapter on the application of the lessons taught us by the great war. Dr. Libby writes with a detailed knowledge of the subject, and his book is a very useful introduction to the study of the evolution of the science and art of medicine.

### CREATURES OF THE WILD.

**Observations and Reflections on Wild Creatures.** By Colonel G. T. K. Maurice, C.M.G., C.B.E. (Drane, Farringdon Street.)

This volume of nature notes is a highly original venture. In the Preface the author disclaims any pretensions to be an expert on Ornithology; but he is no " closet " naturalist. Bred on the Wiltshire Downs, and thereafter a sojourner in many lands to which his profession called him, he is a worshipper of the beautiful as displayed in the works of Nature and an untiring observer. Natural History for him is not merely a hobby, and he here gives us of his experiences and invites us to share his enthusiasm. Of the cut and dried results of other people's observations he will have little or none. Anything that approaches " dogma " is to him *anathema.* And so all through the book there runs a vein of stolid scepticism, an anxiety to record nothing for which he cannot personally vouch, a determination to draw no conclusion from premises not scientifically provable. He writes more as a learner than as a teacher. If he cannot identify a bird or an insect, he does not hesitate to say so; he never guesses at truth. It is, however, to be regretted that he has not made a post-war visit of identification to South Kensington. Most of his unidentified species can be identified there, either in the Bird Gallery or in the collection of birds from the Balkan Front presented by Major Stormonth Darling.

Colonel Maurice's observations are made mostly from the saddle, and he is no doubt correct in supposing that the horse under him tends to disarm suspicion. It is pleasant to ride with him, to listen to his *causeries*, to share his experiences among the local fauna. His great object is to induce you to observe yourself. There is not the least occasion to agree with him; is there not a blank page at the end of every chapter whereon to register refutations or confirmations? Those who want to read of killing will be disappointed; Colonel Maurice has a horror of the collector, and a special loathing for the denizens of the Mediterranean islands and coast who shoot birds on migration. Rather than kill he will leave a rare bird unidentified; it were well that more followed his example. The book was mostly written on service. The roar of guns is audible in the distance; observation is limited by " bounds " and subservient to duty. Three of the longer chapters were delivered, or intended to be delivered, as lectures to men serving in Macedonia. Like the rest of the book, the style is a trifle rugged, and the subject matter inclined to be discursive, as Col. Maurice recognises.

Two of the lectures deal with the problems of instinct and migration. The latter is a favourite topic with the author, and he has much to adduce in support of his theory that bird migration is due primarily to hunger. He adds his quota to the already vast accumulation of existing data, but does not account for the fact that migrants invariably nest at the most northern limit of their migrations. In discussing instinct, Colonel Maurice is not on such sure ground as he could wish. " I assume for the sake of thinking about instinct that creatures, other than man, have mind, will, intelligence, reason, memory. . . . I do not define what these things are. I assume them to be manifestations of properties of nervous matter." This is very much like an excursion into the speculative, a departure from the author's usually Bergson-like anxiety to deduce from nothing but the scientifically provable. If the qualities mentioned are inherent in all nervous matter, how comes it that they are dormant over so large an area of the zoological kingdom! Why can we foretell accurately how an animal, bird or insect will act at given seasons, and under given circumstances, while in the case of men it is impossible? Is not instinct rather a kind of inexorable guidance, a subjection to immutable laws which must be obeyed because those for whom they are made are incapable of breaking them?

But, speculation apart, there is something on nearly every page of the book to stimulate the interest of the man who loves the country. The observations are by no means confined to birds. Badgers; foxes; spiders; the tortoise, its amours and oviposition; snakes; ants, bees and wasps; the dung-rolling beetle and the processionary caterpillar pass in kaleidoscopic view before us, and on each the author has something original to say. The book is, indeed, the work of a genuine enthusiast.

### HAPPY HOMES FOR ALL.

**A National Health Policy.** By Dr. Harry Roberts. (Labour Publishing Co. 3s. 6d.)

On page 50 of this book, Dr. Roberts says:—" It is utterly futile to discuss schemes for the improvement of the National Health unless we are prepared, as a primary consideration, to provide every family in the country with a decent home, affording the minimum conditions in which a healthy life is even possible." That is Dr. Roberts's main theme in this book, in which also he has much to say about the need for organization of medical practice, the co-ordination of health services, the development of panel practice and the importance of birth-control. Elsewhere he says that " the most urgent of all health reforms . . . is the education of the public."

Dr. Roberts is a very busy general practitioner who is not too busy to look about him and appreciate that all his efforts and those of his fellow practitioners, and all the schemes for patching up and curing are of little avail so long as there is so much to be done at the source. He finds time to write in an arresting and informative manner about it all. He has a way of getting a good deal of food for thought into a short sentence as when, *à propos* of overcrowding, he says, " Of the Scottish population, one-quarter lives in Glasgow," or " There is nothing about Whitechapel that makes it peculiarly fitted for tailoring."

Dr. Roberts is of opinion that the thinning out of our populous areas, the construction of new towns and garden cities, the abolition of smoke and the provision of a healthy, sunny environment for everyone is practicable and indeed easy of accomplishment, and we gather that twenty years hence it may be accomplished if the will to do it exists. It is, of course, not fair to expect Dr. Roberts to examine the economic difficulties which bar the way. But they are the gravest part of the problem, and not to be solved by general statements such as those in a recent series of articles by Mr. Philip Snowden, M.P., in the *Morning Post*, in which it is stated vaguely that the money will be found.

Dr. Roberts's book should be read and re-read by those who have the health problems of this country at heart.

### THE DOCTOR IN THE MISSION FIELD.

**Medical Practice in Africa and the East.** Edited by Hugh Martin, M.A. With an Introduction by Stephen Paget. (Student Christian Movement; 4s. net.)

It is the aim of this book to present a true conception of Medical Missionary work by means of various letters written from the mission field. Medical missions need no justification, but they do need better support, and ignorance of the conditions they struggle to remedy no doubt accounts for much of the apathy that exists concerning their work. Here we have some really vivid accounts of the difficulties with which the medical missionary is confronted, and of the multitudes that await the healer who unhappily is often too late to save them. " The crying need of India, Africa and the East," says Dr. Holland of the C.M.S. Hospital, Kashmir, " is a sufficient supply of qualified practitioners, scattered through every small town and district, who are competent to diagnose and send cases to the surgeon before it is too late to operate " :—

> " When one contrasts the scope for surgery at home and abroad there is practically no comparison. Major surgery at home is confined to the favoured few, whereas abroad the young medical missionary may be called upon within a month of landing in the country to perform an ovariotomy or remove an intralaryngeal tumour. The scope in the mission field is so great that one certainly marvels that more do not come out from Britain to work in the well nigh limitless field of surgery the mission field offers. . . . In India the barber was the surgeon and is still in the outlying districts. I might allude to one enterprising oculist who claimed to be a disciple of mine, and promised cure for all leucomas and corneal opacities by excising the portion of cornea involved! I put the District Magistrate on his track; but unfortunately he escaped to ply his trade in other parts."

These letters of devoted men and women, though addressed especially to their medical colleagues at home, should appeal to all who wish to know something of the healing of the nations.

### MISCELLANEOUS NOTICES.

### A Manual for Nurses.

A Manual for Nurses on Abdominal Surgery. By Harold Burrows, C.B.E., F.R.C.S. (The Scientific Press. 4s. net.)—A second edition, rewritten and enlarged, of a valuable practical handbook. The section dealing with shock has been remodelled entirely, and certain ills and their remedies omitted before are now included.

### How to Become a Nurse.

How to Become a Nurse. Revised and Brought Up to Date. By E. Margaret Fox, R.R.C. (Scientific Press; 4s. net.)—This is the tenth edition of a very useful book of reference, originally edited by the late Sir Henry Burdett. In addition to full particulars of training conditions in every kind of institution in the United Kingdom and the Colonies, the syllabus of the General Nursing Council is given, and there is a chapter which will be eagerly read by all would-be probationers on " Advice to Women Desirous of Becoming Nurses." Most girls have the vaguest notion of the way to set about entering a hospital or of what becoming a nurse really entails. This chapter tells them clearly what lies before them with no minimising of difficulties and with plenty of practical suggestions. There is also a chapter on the Training of Male Nurses, and no detail seems to have been forgotten in a particularly well-compiled handbook.

### The Work of Guy's.

Guy's Hospital Reports. Vol. 73. No. 2. April, 1923. —The current number (issued long after the date it bears) contains many attractive features, but one which will particularly appeal to those long associated with hospital life is an admirably written and illustrated memoir of Sir Frederick Taylor. On the purely medical side several subjects already taken in hand by various workers in the hospital receive further attention. The Editor, Dr. A. F. Hurst, has wisely adopted the principle of publishing collateral comments upon this work, but made by others than those directly connected with modern Guy's. Thus one reads contributions from Dr. Adolphe Abrahams of the Westminster, Professor Harvey Cushing of Harvard University, and Dr. Wm. Hunter of Charing Cross Hospital running parallel with Guy's own records. These latter include publications from the Biochemical Department by Dr. J. H. Ryffel, observations on intracranial aneurysms by Dr. C. P. Symonds, further excellent studies in tumour formation by Dr. G. W. Nicholson, a laboratory investigation as to the value of urotropine as a biliary antiseptic by Dr. F. A. Knott, a commentary on the etiology of varicose veins by Mr. Philip Turner and observations from the obstetrical department by Mr. Frank Cook. The whole is printed clearly and in admirable style, making this number a worthy successor of the many excellent Reports which preceded it.

### A Text Book of Nutrition.

Nutrition of Mother and Child. By C. Ulysses Moore, M.D., M.Sc. (Ped.), Instructor in Diseases of Children, University of Oregon Medical School, etc. (Lippincott. 8s. 6d. net.)—This book contains some very sound teaching on the subject of nutrition, and is not too abstruse to appeal to the young mothers, social workers and nurses for whom it is specially written. Its purpose, to which the author firmly adheres throughout the thirteen chapters, is to teach mothers how to render their families less subject to disease by proper care and diet, and to this end due emphasis is placed upon the value of breast feeding, vitamines and the mineral content of the diet. The language is as untechnical as possible, and even the chapter on vitamines is so written as to make really attractive reading for the unlearned. Rickets with its attendant evils is plainly and simply explained, and the illustrations of this and other diseased conditions are excellent. Diet during pregnancy and lactation receives much attention, and, allowing for national differences in foods, the specimen diet-table shown is as well suited to the needs of an English, as of an American woman. The chapters on children's diets are also extremely sensible, and their value is increased by being supplemented by a series of recipes for simple dishes, contributed by Myrtle Josephine Ferguson, B.S., Professor of Nutrition in the Iowa State College.

